# Protecting Biodiversity when Money Matters: Maximizing Return on Investment

**DOI:** 10.1371/journal.pone.0001515

**Published:** 2008-01-30

**Authors:** Emma C. Underwood, M. Rebecca Shaw, Kerrie A. Wilson, Peter Kareiva, Kirk R. Klausmeyer, Marissa F. McBride, Michael Bode, Scott A. Morrison, Jonathan M. Hoekstra, Hugh P. Possingham

**Affiliations:** 1 Department of Environmental Science and Policy, University of California Davis, Davis, California, United States of America; 2 The Nature Conservancy, San Francisco, California, United States of America; 3 The Ecology Centre, School of Integrative Biology, University of Queensland, Brisbane, Australia; 4 The Nature Conservancy, Seattle, Washington, United States of America; 5 Environmental Science, School of Botany, University of Melbourne, Melbourne, Australia; University of Pretoria, South Africa

## Abstract

**Background:**

Conventional wisdom identifies biodiversity hotspots as priorities for conservation investment because they capture dense concentrations of species. However, density of species does not necessarily imply conservation ‘efficiency’. Here we explicitly consider conservation efficiency in terms of species protected per dollar invested.

**Methodology/Principal Findings:**

We apply a dynamic return on investment approach to a global biome and compare it with three alternate priority setting approaches and a random allocation of funding. After twenty years of acquiring habitat, the return on investment approach protects between 32% and 69% more species compared to the other priority setting approaches. To correct for potential inefficiencies of protecting the same species multiple times we account for the complementarity of species, protecting up to three times more distinct vertebrate species than alternate approaches.

**Conclusions/Significance:**

Incorporating costs in a return on investment framework expands priorities to include areas not traditionally highlighted as priorities based on conventional irreplaceability and vulnerability approaches.

## Introduction

Current rates of biodiversity loss are unprecedented in the history of the Earth [1]. In response to the accelerating extinction of species, international conservation organizations have generated global biodiversity conservation templates [2] as strategic guides to conservation investment. High-profile global scale priority templates include Hotspots [3], the Global 200 [4], Endemic Bird Areas [5], and Crisis Ecoregions [6]. The purpose of these approaches is to guide the allocation of funding to areas where there is both the greatest need and greatest biodiversity payoff [3].

Implicit in these templates for conservation focus is the notion of efficient investment through prioritization of geographies. Some approaches, such as Hotspots, make explicit claims of efficiency - protection of 1.4% of the earth's surface would capture 44% of vascular plants and over a third of all vertebrate species [3]. However, minimizing the area protected, spatial efficiency, does not necessarily translate into cost efficiency. In a world with limited conservation funds [7], efficiency would be better measured in terms of conservation return on financial investment, such as the number of species protected per dollar expended over a fixed amount of time.

It is increasingly recognized that including the economic costs of conservation to maximize the greatest return on investments can lead to substantially larger biological gains [8]. We present an application of the return on investment approach across a global biome to identify investment priorities for protecting both a greater total number of species as well as more distinct species overall. Central to this approach is the explicit inclusion of costs in determining where to allocate scarce conservation dollars to meet a specific conservation objective and the consideration of how investment should change through time to meet this objective [9]–[11]. Specifically, we first evaluate the performance of the return on investment approach as a global priority setting technique with three alternate approaches which emulate existing priority setting methods Endemic Bird Areas [5], Crisis Biomes [6], the cost of protecting threatened vertebrates [12] and finally, an extreme of no priority setting method at all, the random allocation of conservation resources. Second, in recognizing that funding allocation will benefit many of the same species in multiple ecoregions, we demonstrate the incorporation of species complementarity (the extent to which new protected lands add species not previously protected) within a dynamic resource allocation framework. Using the return on investment approach with an objective of protecting not only endemic vertebrate species but also the complement of non-endemics [13] we can realize even greater efficiency in the biodiversity returns per dollar spent (see Text S1 and Fig. S1).

**Figure 1:**
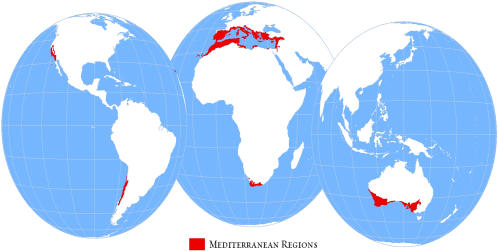
Global distribution of the mediterranean biome [22] which lies within the countries of South Africa, Chile, Australia, the United States of America, Mexico, Portugal, Spain, France, Italy, Monaco, Slovenia, Croatia, Bosnia and Herzegovina, Serbia, Montenegro, Macedonia, Albania, Greece, Turkey, Cyprus, Malta, Syria, Lebanon, Israel, Gaza Strip, West Bank, Iraq, Jordon, Egypt, Libya, Tunisia, Algeria, Morocco and Western Sahara.

We focus on a global biome that has consistently emerged as a focus for global biodiversity conservation [2] - *mediterranean forests, woodlands, and scrub*. The mediterranean biome occurs in California and Mexico, Chile, South Africa, Australia, and the entire Mediterranean Basin including northern Africa and the Near East, spanning a broad social, political, and economic spectrum (Fig. 1). Mediterranean regions are renowned for both their high levels of endemic biodiversity [14], [15] and high vulnerability [16], [17]. As such, they have been designated as crucial to global biodiversity conservation efforts by a number of priority setting approaches [3], [4], [6]. With less than 5% of the mediterranean biome protected [6], efficiently expanding the protected areas network is central to achieving global conservation objectives [18]. We illustrate a return on investment approach for deciding the sequence of investment in different mediterranean ecoregions.

A number of previous studies have incorporated costs into algorithms to determine ranking schemes for candidate sites within a static framework. Ando et al. (1998) produced a list of counties in the United States that would capture the most endangered species per dollar. More recently, studies have included costs within a dynamic framework. Costello and Polasky (2004) identified conservation priorities in southwest California which accounted for the risk of future land development. Other return on investment applications have expanded beyond simply land acquisition to include the cost of other conservation actions to abate threats, such as restoration and control of invasive species [19].

Wilson et al. (2006) determined priorities for protecting endemic birds considering the annual rate of forest loss and diminishing returns as investments proceed. Other studies have found that including the availability of reserves, or ability to swap reserves for more favorable ones, in the future can increase the efficiency of conservation [20], [21]. Our approach is similarly dynamic and extends these studies to consider current protection, amount of land converted, amount of habitat available, and data on endemism and the total richness of both plants and vertebrates. We also incorporate species complementarity into priority setting at the large scale, where partial rather than entire planning units can be acquired. The dynamic return on investment framework we present can be used to address resource allocation problems that conservation organizations face from scales ranging from sub-ecoregional units to global analysis.

## Methods

The return on investment approach seeks to maximize the return (defined in units of a clearly defined objective) per unit investment (e.g., dollars, people, time) [19]. The return reflects the objectives of the funding organization and could include multiple factors, such as species richness, the value of ecosystems services or recreational opportunities. Given, the limited resources available for conservation combined with lack of current protection in the mediterranean biome, we seek the greatest return from each dollar invested for protecting biodiversity and utilize the number of species protected as our return.

The spatial units of analysis consist of the 39 ecoregions within the mediterranean biome [22]. Within each ecoregion we articulate five factors. First, the number of plant and vertebrate species in each ecoregion that could benefit from protection were compiled from existing global datasets organized by ecoregion [23], [24]. This permits us to compare the performance of different priority setting approaches using five simple measures of biodiversity value: total species richness, plant richness, vertebrate richness, endemic vertebrate richness and threatened species richness (IUCN Red List categories critical, endangered, and vulnerable). Second, the area currently protected was calculated based on the World Database on Protected Areas IUCN classes I–IV (2006). Since less than one third of the protected areas in the database have associated polygon data we used the geographic point data. If a protected area overlapped with more than one ecoregion the extent of that protected area was assigned to the ecoregion that the point fell within. Third, the cost of acquiring land for protection was calculated by applying an equation based on a study of 139 terrestrial conservation programs worldwide [25] (see Text S1). Fourth, the amount of natural and semi-natural area available for protected area acquisition was calculated based on global land cover data [26]. Fifth, the projected rate of habitat loss was calculated by combining areas of high human impact based on land use, population pressure, infrastructure and access [27] with the projected 2015 population data. Highly impacted areas were identified based on cross-referencing the Human Footprint data with country scale data across the mediterranean biome on the extent of urban and agricultural area and normalized by population data for the year 2000. To predict future impacts to habitat, the number of highly impacted gridcells per ecoregion were multiplied by the projected population growth rate between 2000 and 2015 to provide a per annum rate of future impact [28] (see Text S1 and Table S1).

We utilize a return on investment ‘maximize gain heuristic’ similar to the one described in Wilson et al. (2006). The maximize gain heuristic directs investment at each timestep to the ecoregion where the most species can be protected given a fixed budget (see Text S1). We assume that the biodiversity benefit with increasing investment is represented by the species-area relationship [11], [29].

To assess the relative effectiveness of our approach as a global priority setting technique, we compare the number of species protected using return on investment after 20 years with the number protected when the annual budget is allocated proportional to other characteristics of the ecoregions at the outset of funding. These alternate approaches are also conducted within a dynamic framework accounting for the change in habitat available each year (informed by the predicted rate of habitat loss and changes in the amount of protection with investment) but, with the exception of (c), do not explicitly consider cost efficiency (see Text S1):

- Areas with endemic species (hereafter Endemism): funding is allocated proportional to the number of endemic vertebrate species in each ecoregion paralleling the Endemic Areas approach [5];
- Crisis Biomes: funding is allocated proportional to an index which reflects the percent habitat converted divided by the percent habitat protected in each ecoregion [6];
- Areas with high threatened species per dollar (hereafter Threatened species/dollar): funding is allocated proportional on an index which reflects the number of threatened species divided by the cost of land in each ecoregion (to reflect key elements of Ando et al.[12]); and
- Random - each year the annual budget is allocated to any number of the 39 ecoregions at random.

When incorporating the complementarity of species within the return on investment framework, our objective is to protect as many different, or ‘distinct’, species as possible. To account for complementarity within biogeographic realms which contain more than one ecoregion, we consider both the number of species endemic to each ecoregion and also those occurring across multiple ecoregions. Both of these are represented by a separate species area curves. As when prioritizing based on species richness or endemism alone, the objective when using complementarity is to maximize the biodiversity benefit per dollar invested. Investment thus favors ecoregions where the sum of the species protected across all of the species area curves is greatest (see Text S1 and Fig. S1). Ideally, owing to the renown plant diversity of mediterranean regions [14], our application of complementarity would seek to maximize the number of distinct plant species across ecoregions. However, since lists of vascular plants by ecoregion are currently unavailable we demonstrate our approach using vertebrate data.

In applying our return on investment approach we constrain conservation efforts by a budget of $100 million per year for a period of 20 years. We also assume that investment results in immediate and successful protection of species.

## Results

### Biodiversity Benefit

**Figure 2:**
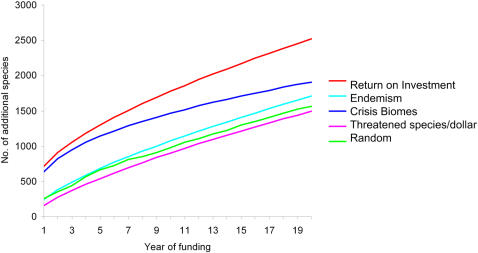
Comparison of the number of additional species (plants and vertebrates) protected after 20 years in the mediterranean biome using return on investment and five alternative approaches with an annual budget of $100 million per annum.

After 20 years, the biodiversity benefited by the return on investment approach is consistently greater than with the four alternate approaches. Return on investment protected 32% to 69% more plant and vertebrate species, 2 to 5 more endemic vertebrates, and 4 to 6 more threatened vertebrates (Table 1, Fig. 2). The second most effective method for protecting total species richness, vertebrate and plant species richness was the Crisis Biomes approach with funding allocated according to an index based on the amount of conversion and protection in each ecoregion, although return on investment still protects a third more total species and five more endemic vertebrates. Even in comparison to an approach specifically designed to capture endemic species (Endemism) performance is again suboptimal by comparison with return on investment protecting two more endemics. Although this is a small number after 20 years, the comparative benefits associated with return on investment will continue to increase over time. In comparison to the Threatened species/dollar approach, which prioritizes ecoregions where the cost of protecting threatened species is lowest, return on investment protected five more threatened species and four more endemic species.

**Table 1:** Number of additional species protected for five measures of biodiversity by applying a return on investment approach and four alternative priority setting approaches with a conservation budget of $100 million per year over 20 years.

Prioritization approach	Total species richness	Vertebrate species richness	Plant species richness	Endemic vertebrate richness	Threatened vertebrates	No. of distinct vertebrates
Return on Investment	2524	341	2231	8	14	69
Areas with endemic species	1705	201	1504	6	8	45
Crisis biomes	1910	227	1683	3	10	23
High threatened species per dollar	1495	218	1276	4	9	43
Random	1571	208	1383	3	8	27

There are 1,545 distinct vertebrate species captured within the current protected areas network in mediterranean ecoregions. When return on investment considered complementarity, 69 additional distinct vertebrate species were protected, i.e., species which are endemic to an ecoregion and those which occur across multiple ecoregions within the five biogeographic realms. Endemism and Threatened species/dollar performed next well with 45 and 43 distinct species respectively, while allocating the budget randomly captured more distinct vertebrates [27] than the Crisis Biomes approach [23].

### Investment schedule

In contrast to static templates of conventional approaches of identifying priorities the return on investment approach delivered a temporal roadmap for allocating funds to ensure investment efficiency into the future. To protect total species richness, the return on investment approach delivered an investment schedule which focused funds on just 12 of the 39 ecoregions (Table 2). Funds were initially invested in two Mediterranean Basin ecoregions - the *Corsican montane broadleaf and mixed forests* and the *Mediterranean acacia-argania dry woodlands and succulent thickets*, spanning northwest Africa and the two eastern Canary Islands. Both ecoregions were prioritized for investment owing to extremely low levels of current protection and the cost efficiency of protecting additional species. In years 4 to 8, investment extended outside the Mediterranean Basin to include the *Lowland fynbos and renosterveld* of South Africa, the *Swan Coastal Plain* of Australia, and the *Chilean matorral*. After 20 years, conservation funds were invested in four of the five mediterranean regions: 35% of land acquired globally is in South Africa, 18% in Australia, 10% in Chile, and 36% in the Mediterranean Basin.

**Table 2:** Priority ecoregions within the mediterranean biome using a return on investment approach with an annual budget of $100 million over 20 years.

Ecoregion Name	Swan Coastal Plain Scrub and Woodlands	Mount Lofty woodlands	Naracoorte woodlands	Albany thickets	Lowland fynbos and renosterveld	Chilean matorral	Anatolian conifer and deciduous mixed forests	Canary Islands dry woodlands and forests	Corsican montane broadleaf and mixed forests	Crete Mediterranean forests	Eastern Mediterranean conifer-sclerophyllous-broadleaf forests	Mediterranean acacia-argania dry woodlands and succulent thickets	Mediterranean dry woodlands and steppe	Mediterranean woodlands and forests	Southeastern Iberian shrubs and woodlands	Southern Anatolian montane conifer and deciduous forests
Region	Aus	Aus	Aus	SA	SA	Chl	MedB	MedB	MedB	MedB	MedB	MedB	MedB	MedB	MedB	MedB
Area (km^2^)	15,210	23,786	27,531	17,135	32,764	148,383	86,382	4,968	3,633	8,193	143,882	99,985	292,082	358,226	2,849	76,449
Projected habitat loss (%yr^−^1)	0.0097	0.0093	0.0038	0.0019	0.0000	0.0081	0.0090	0.0058	0.0061	0.0000	0.0204	0.0075	0.0035	0.0106	0.0187	0.0116
% converted	46.9	67.3	60.7	6.8	32.1	16.3	43.7	2.3	0.7	16.6	29.4	24.0	2.0	26.5	56.4	25.5
% protected	8.7	6.8	5.5	8.2	3.6	0.9	0.6	4.5	0.0	0.7	0.5	0.1	1.0	0.5	2.4	1.4
Cost (1,000 US$km^−2^)	$229	$229	$229	$188	$188	$174	$358	$1,375	$2,149	$1,096	$476	$182	$93	$124	$1,375	$407
**Total Rich:** % budget	12%	3%		2%	22%	6%	7%		15%	9%	4%	12%			0.1%	8%
order funded	5	10		11	4	7	6		1	3	9	2			12	8
sp with investment	5.33	3.74		3.68	7.94	4.69	5.24		264.08	9.56	4.06	36.27			3.35	4.31
**Pl Rich:** % budget	12%	2%			21%	8%	7%		16%	10%	4%	11%				10%
order funded	5	10			4	7	6		1	3	9	2				8
sp with investment	4.61	3.12			6.87	4.34	4.58		241.42	8.72	3.49	29.87				3.93
**V Rich:** % budget	9%	5%	4%	16%	18%		4%		8%	3%	3%	14%	1%	5%	11%	
order funded	7	10	11	5	4		9		1	6	12	2	13	8	3	
sp with investment	0.72	0.61	0.60	0.95	1.07		0.66		22.67	0.84	0.57	6.40	0.53	0.66	1.35	
**End V Rich:** % budget					14%	44%		19%	3%	2%		12%		6%		
order funded					4	3		5	1	7		2		6		
sp with investment					0.02	0.04		0.02	0.27	0.02		0.13		0.02		
**V Comp:** % budget				6%	13%	32%			0.1%			8%	22%	19%		
rank				7	6	3			1			2	5	4		
**Overall Average % Budget**	**6%**	**2%**	**1%**	**5%**	**18%**	**18%**	**4%**	**4%**	**8%**	**5%**	**2%**	**11%**	**5%**	**6%**	**2%**	**4%**

The ‘order funded’ shows the ecoregion's rank for investment using the return on investment approach. This order reflects the number of incremental species gained with initial investment (‘sp with investment’) calculated based on allocating all funding to each ecoregion in the first time step (see supporting information). Codes are as follows: Aus = Australia, SA = South Africa, Chl = Chile, and MedB = Mediterranean Basin and biodiversity measure codes are: Pl = Plant, V = Vertebrate, Rich = Richness, End = Endemism, Comp = Complementarity

When the complementarity of vertebrate species is considered, initial investment continued to fund the same two ecoregions in the Mediterranean Basin as when priorities are set using total species richness. However, investment in these ecoregions accounted for much less of the total budget after 20 years: 0.1% compared to 15% for the *Corsican montane forests* and 8% compared to 12% for the *Mediterranean acacia-argania dry woodlands and succulent thickets* ecoregion. A third of the budget, higher than using any other biodiversity measure, was then allocated to the *Chilean matorral*. After 20 years, land was acquired in three of the five mediterranean regions (60% of the land protected globally is in the Mediterranean Basin, 26% in Chile, and 14% in South Africa). Investment in California-Mexico did not occur until year 28 - directed to the *California chaparral and coastal sage* ecoregion.

Some ecoregions were consistently prioritized regardless of the biodiversity measure, e.g., the *Lowland fynbos and renosterveld*, while others are selected based only on a single measure such as the *Southeastern Iberian shrublands and woodlands* in the Mediterranean Basin or the *Naracoorte woodlands* in Australia. The average percent of the budget received across all biodiversity measures revealed that certain ecoregions received a comparatively large amount of the budget. For example, the *Lowland fynbos and renosterveld* and the *Chilean matorral* both received an average of 18% by year 20, indicating their important contribution to species protection regardless of biodiversity benefit used (Table 2).

## Discussion

**Figure 3:**
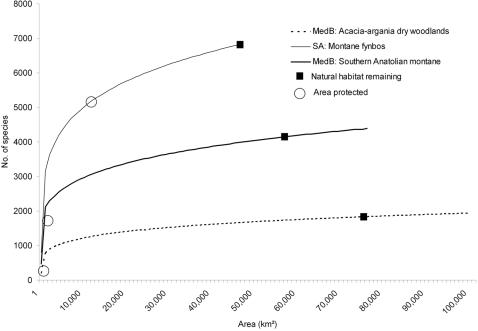
Comparison of species area curves for three ecoregions using total species richness data. Curve end points represent the total area and number of species for each ecoregion, e.g., 76,449 km^2^ and 4,387 species for MedB: Southern Anatolian montane. Factors informing the return on investment approach include; (i) the amount of ecoregion currently protected (○); (ii) available natural habitat indicated by the region between the area protected (○) and the area converted (▪), and the steepness of the curve at this point; and (iii) the cost of land (indicated by line weights). Budget allocation is based on a combination of the number of species protected and the cost of land (by multiplying the area protected by its cost per unit area to generate a species-investment curve). Low existing protection combined with the cost efficiency of protecting species prioritizes investment in the MedB: Acacia-argania dry woodlands (1). The relatively high potential returns from investing in the MedB: Southern Anatolian montane ecoregion (2) gives it a higher investment priority than the SA: Montane fynbos ecoregion (3) although the cost of conservation is double, while the latter ecoregion receives funding after 40 years. Full region and ecoregion names listed in Table 2.

After 20 years, the return on investment approach consistently protected substantially higher numbers of species than do alternate approaches, no matter what the biodiversity measure (Fig. 2). The Crisis Biomes approach performed well initially as funding allocation prioritized ecoregions with low levels of protection thereby capturing high numbers of species. For example, similar to return on investment the *Corsican montane forests* were a priority owing to protection levels of <1%. The other approaches did not explicitly consider existing protection thus overlook capturing high numbers of species associated with currently under-protected ecoregions (Fig. 3). In the Endemism approach funding is inefficiently directed to already well protected ecoregions such as the *Esperance mallee* ecoregion in Australia (22% protection) or the *Montane fynbos and renosterveld ecoregion* (24% protection).

In contrast, our return on investment approach identified an economically efficient suite of conservation priorities by accounting for key factors not included in conventional priority setting approaches, thereby maximizing the efficient use of limited conservation dollars. Although biodiversity varies substantially across the 39 mediterranean ecoregions (total species richness ranges between 668 and 6,805 species and endemic vertebrate richness varies between 0 and 25 species) costs vary far more - from $93,500 to $2,500,000 per km^2^ (Table S1). The disparity in costs means there is great potential for efficient financial investment. However, cost alone does not drive the outcome - it is the ratio of cost to the biodiversity benefited from conservation and the current level of investment in protection that is critical (Table 2). The return on investment approach assigns funding based on a review of conservation status at a specified interval and using that information allocates investment to ecoregions that provide the greatest returns (Fig. 3). Prioritizations that neglect cost, implicitly assume that this factor is the same everywhere – if this assumption is violated, then any claim of efficiency is unsupported.

However, our comparison of the number of species (plant, vertebrate and total species) captured by any approach are potentially inflated since the same species may be protected in multiple ecoregions. A better comparison might be to focus on the results of the number of endemic species protected (Table 1) - particularly appealing since it has been found to roughly correlate with total species richness in some cases [30], [31]. Nonetheless, protecting species endemic to one ecoregion alone is inefficient and suboptimal as it disregards information on species that are found in multiple ecoregions [32]. We therefore incorporated species complementarity within the dynamic return on investment framework and identified seven mediterranean ecoregions in three biogeographic realms which are priorities for future investment based on maximizing the number of distinct vertebrates. Realms where there is a high degree of overlap between vertebrates in different ecoregions, such as mediterranean region of Australia, were not considered priorities for conservation over the next 20 years. Our approach builds on previous work that has included species complementarity into reserve design at the small scale to account for the overlap in species ranges and the acquisition of partial rather than entire planning units. This approach not only increases investment efficiency but permits priority setting to consider the number of distinct species rather than simply tradeoff between priorities identified based on species richness or endemic species richness metrics.

For prioritizing conservation efforts over practical timeframes, adoption of the return on investment approach will protect more total numbers of species within each ecoregion as well as more distinct species overall. Well-known biodiversity hotspots are selected - such as the *Chilean matorral* which harbors the highest number of endemic vertebrates in the mediterranean biome and South Africa's *Lowland fynbos and renosterveld ecoregion* with high plant species richness. However, the highest return on conservation investment is achieved if funding is also directed to lower-profile ecoregions in Northwest Africa and the Near East. While these ecoregions may not have the highest mediterranean biodiversity (Table S1), the fact that they are poorly conserved at present with low land costs make them conservation priorities owing to the high potential returns from investment. The fact that return on investment encourages conservation investment in places not traditionally recognized as priorities indicates it is of practical, as well as theoretical, importance. Priority ecoregions identified in this study are clearly dependent on the data used for the analysis - cost and rate of habitat loss data could be improved upon, for example, by using regional data. In addition, further refinement of priorities within the mediterranean biome requires lists of vascular plants by ecoregion to allow complementarity to be run on one of the fundamental characteristics of mediterranean regions.

The return on investment approach presented here is demonstrably more efficient than alternate prioritization approaches. Return on investment captured the most species using any biodiversity measure in just over a third of the mediterranean ecoregions compared to alternate methods which allocated funding to most ecoregions, resulting in a conservation plan which is logistically and implementationally impractical. This framework can be applied to any terrestrial biome or spatial scale. Future applications of the return on investment framework could modify the return of the investments. Our objective was to maximize species richness and endemic species richness however, alternate or multiple returns such as the value of ecosystem services or recreation opportunities could be used. Moving beyond a strictly species focus is advantageous, however, it still does not reflect the broader concept and complexities of critical natural capital - i.e., the irreplaceable functions the natural environment performs - both from an ecocentric perspective (ecosystems maintaining environmental health) and anthropocentric perspective (ecosystem services that are important for human survival and well-being) [33], [34]. The framework could also be improved by systematically incorporating factors beyond cost, such as the ability to leverage funding. Conservation investments for protecting species might be matched by funds from within that region or, alternatively, spending for conservation could leverage funds for non-species objectives, such as developing markets for ecosystem goods and services, underlining the multi-criteria framework that conservation spending exists within. Similarly, future modifications could incorporate the risk of investment failure. Since investment decisions are often judged on their capacity to sustain the value of those investments, the approach should also incorporate the risk that investments might be squandered if conservation agreements are violated or social or political instability leads to habitat destruction [35]. The return on investment approach can and should be expanded to integrate these additional factors - the only limitation is in identifying and obtaining quantitative data expressing leverage potential or risk of investment failure. Even without such improvements, however, return on investment advances conventional global and country priority setting approaches by incorporating costs and diminishing returns to provide a dynamic spatial and temporal roadmap for protecting species more efficiently in a rapidly changing world.

## Supporting Information

Text S1 Detailed description of methods and data
(0.07 MB DOC)

Table S1 Characteristics of the 39 mediterranean ecoregions: total ecoregion area, projected habitat loss (%yr-1), area of natural and semi-natural habitat, area converted and area protected, cost (US$) per km-2, and information on plant species richness (6), vertebrate (‘Vert’) species richness and endemic richness (9). The number of threatened vertebrate species (IUCN Red List categories critical, endangered, and vulnerable) omits the three extinct vertebrate species in the database. Region codes are as follows: Aus = Australia, Ca&Mex = California and Mexico, SA = South Africa, Chl = Chile, and MedB = Mediterranean Basin.
(0.15 MB DOC)

Figure S1 A hypothetical example of the species area curves required to account for complementarity for a biogeographic realm that contains two ecoregions (A and B). Curve A and curve B represent the number of endemic species contained in the respective ecoregions A and B and curve AB represents overlapping species in ecoregions A and B. The circles (O) indicate the amount of area currently protected in each ecoregion.
(0.44 MB DOC)
