# Supplementary material for: Protecting Biodiversity when Money Matters: Maximizing Return on Investment
Source: PLoS One. 2008 Jan 30;3(1):e1515. doi: 10.1371/journal.pone.0001515 (PMC2212107; doi:10.1371/journal.pone.0001515)
Supplement: Text S1 — Detailed description of methods and data (0.07 MB DOC) [file pone.0001515.s001.doc]

## 1. Area available for conservation

The area of natural and semi-natural vegetation within each ecoregion was calculated using a fusion of two versions of the 1 km resolution Global Land Cover (GLC) coordinated by the Joint Research Centre in Italy (<http://www-gvm.jrc.it/glc2000/>) (5). The most recent version, GLC 2004, (version 1.1) was cross-referenced with regional data and visual inspection showed notable improvements to GLC 2000 (version 1) in some areas, e.g., the Near East and the Canary Islands. However, GLC 2004 appears to be less accurate in California; according to this version, only 2.8% the California interior chaparral and woodlands ecoregion is converted to urban and agricultural uses. In contrast, GLC 2000 indicates this ecoregion is 12.8% converted, which more closely resembles the amount converted according to regional land cover sources (13.2%) (2). Consequently, area estimates for California and Mexico are generated from GLC 2000 and for the other four regions we use GLC 2004.

2. Annual Rate of Habitat Loss

To calculate the annual rate of habitat loss, we used the human footprint data, which provides an index (normalized) ranging from 0 (no impact) to 100 (high impact) for each gridcell on the earth (8). We first selected a minimum threshold index value (25) based on cross-referencing with urban and agricultural areas in regional scale datasets to map areas that are highly impacted. To predict future impacts to habitat, the number of highly impacted gridcells per ecoregion were multiplied by the projected population growth rate between 2000 and 2015 to provide a per annum rate of future impact. Projected population growth rates for 2015 were taken from the Gridded Population of the World (3). In areas with a predicted drop in population, we assume that habitat is unlikely to improve in these ecoregions, so all negative growth rate values were converted to zero. (Table S1).

*For example;* assume ecoregion X has 1,000 km^2^ of highly impacted land (land with a human footprint index value greater than 25). This ecoregion had a population of 200,000 in 2000 and a projected population of 230,000 in 2015. This implies an average population growth of 2,000 people per year, or 1% of the 2000 value. When this rate is multiplied by the area of highly impacted land in 2000 (1,000 km^2^), the result is the average annual amount of habitat loss (100 km^2^). This amount is divided by the land available for protection in the ecoregion to give the rate of loss of habitat per year. In the return on investment algorithm, the *rate* of loss is constant over time, but the *amount* of land available for protection decreases each year, so the *amount* of habitat lost also decreases.

3. Cost of Land

To estimate the cost of purchasing land in each ecoregion we applied an equation developed by Balmford et al. (2003) (1) based on a study of 130 terrestrial programs worldwide. The recurrent cost of annual management in US$ km^-2^ is:

log(Cost US$) = 1.61 + 0.57 * log(GNI US$ km^-2^) - 0.7 * log(PPP) - 0.46

* log(Area, km^2^))

We used the average size of protected areas in the mediterranean biome (44 km^2^) as the area term in the equation. Gross National Income (GNI) was compiled from the International Monetary Fund’s International Financial Statistics (2004) and Purchasing Power Parity (PPP) and GDP deflators were acquired from the World Bank (<http://devdata.worldbank.org/wdi2006/contents/Section4.htm>). Note that the PPP term is the PPP conversion factor divided by the exchange rate. To account for ecoregions that span multiple countries we calculated the area-weighted average after we calculated the costs for each country. Countries missing data (e.g., Iraq, Serbia) were omitted from the area-weighted calculations.

Land acquisition costs have been related to recurrent annual management costs (1): for a sample of 19 countries, the national mean cost of land acquisition per km^2^ were on average 50.6 (±13.5) times the national mean recurrent management costs. We multiplied the management costs by 50.6 to estimate the cost of land acquisition in each ecoregion (Table S1).

4. Details on Return on Investment Approach

We utilize the return on investment maximize gain heuristic similar to the one described in Wilson et al. (2006), but treating land as a continuous quantity (i.e., any amount of land can be acquired) rather than a finite set of parcels (i.e., no fractions of parcels can be acquired) and using a higher resolution discretization of time. The maximize gain heuristic directs investment at each timestep to the ecoregion where the most species can be protected given a fixed budget. Each timestep is approximately 1/20^th^ of a year, so the budget allocated in each timestep is approximately $5 million ($100 million / 20). To illustrate this behavior, we present the incremental species that can be protected in the first timestep (IS_j_^P^(1) ) in Table 2 labeled number of ‘species with investment’. The calculation of incremental species is based on the species area-relationship:

$$S_{j}=\alpha_{j}*A_{j}^{Z}$$

where S_j_ is the total number of species (plants, vertebrates, or endemic vertebrates) in ecoregion j, A_j_ is the area of the region of interest (total area of ecoregion) j, and z is 0.2. The constant α_j_ is calculated for each ecoregion by filling in the values for A_j_ and S_j_ in the above equation and solving for α_j_. The initial number of species protected in each ecoregion (S_j_^p^(0) ) is calculated based on the existing amount of protected area (A_j_^P^(0) ):

$$S_{j}^{P}\left(0\right)=\alpha_{j}*A_{j}^{P}\left(0\right)^{Z}$$

The total area that could be protected in the first timestep if the entire budget is allocated to ecoregion j (A_j_^P^(1)), is calculated based on the cost of land in that ecoregion (C_j_), and the total budget for that timestep (B(1)):

$$A_{j}^{P}\left(1\right)=A_{j}^{P}\left(0\right)+\frac{B\left(1\right)}{C_{j}}$$

The total species in timestep 1 that could be protected by allocating all of the budget to ecoregion j is calculated as follows:

$$S_{j}^{P}\left(1\right)=\alpha_{j}*A_{j}^{P}\left(1\right)^{Z}$$

The incremental species (IS_j_^P^(1) ) is the difference between the initial number of species protected and the number of species protected in timestep 1:

$$IS_{j}^{P}\left(1\right)=S_{j}^{P}\left(1\right)-S_{j}^{P}\left(0\right)$$

The maximize gain heuristic calculates the incremental species for all ecoregions for a given budget at each timestep, and then invests in the ecoregion where the incremental species is the greatest. The number of incremental species presented in Table 2 represents the value in the first timestep, but this will change in each subsequent timestep as additional land is protected and species are protected. While this value is only valid for the first investment decision, the rank of the value does correspond with future investment decisions over 20 years because there is little change in the species-investment curves relative to each other.

To assess the performance of the return on investment approach we compared the biodiversity benefit using return on investment with the biodiversity benefit using four alternate approaches and a random allocation of funding. Allocation proportional to these alternate approaches was also conducted within a dynamic framework accounting for the change in habitat available each year *(4;* 7) - informed by the predicted rate of habitat loss and changes in the amount of protection with investment.

5. Incorporating Complementarity in Return on Investment

To incorporate complementarity within biogeographic realms with more than one ecoregion, the biodiversity benefit is represented by separate species-area curves - the number of species occurring across multiple ecoregions is considered independent to those that are endemic to an ecoregion. For example, for a biogeographic realm containing two ecoregions A and B, we consider not only an ‘A species-area curve’ and a ‘B species-area curve’ for species endemic to ecoregions A and B respectively, but also an ‘AB curve’ for the species found in both ecoregion A and ecoregion B (Fig. S1). The AB curve is longer than the curves of the individual ecoregions since it reflects the combined area of the two ecoregions. If the current network of protected areas conserves 100 distinct species in a biogeographic realm containing two ecoregions, this number reflects the endemic species currently protected in curve A (at the circle in Fig. S1) + endemic species currently protected in curve B (circle) + species overlapping in curve AB (circle).

The species area constant alpha for the combined curve (AB) is then calculated as:

$\alpha_{AB}$ = $\frac{S_{AB}}{(A_{A}+A_{B})^{z}}$**,**

Where $S_{AB}$ is the total number of overlapping species and$A_{A}$, $A_{B}$ are the total area of regions A and B respectively. For biogeographic realms containing three or more ecoregions, we consider the species which overlap between component ecoregions: for ecoregions A, B, and C, there are a total of seven species-area curves: A, B, C, AB, AC, BC, and ABC. The number of species-area curves thus increases combinatorially as the number of ecoregions within a realm increases (Fig. S1).

As with prioritizing based on species richness or endemism alone, our objective is to maximize the biodiversity benefit per dollar invested. Consequently, using complementarity with a return on investment objective favors acting in ecoregions where for each dollar invested, the sum of the additional species that would be protected across all of the species-area curves is greatest. For example, the benefit of investing in ecoregion A, is the sum of the incremental species protected across all of the curves that ecoregion A contributes to. The distinction with using the complementarity algorithm is in how the benefit of investing in each ecoregion is calculated, i.e., it uses all of the curves to reflect the complementarity between ecoregions in terms of the species they contain. The amount of area protected on curve AB reflects the sum of area protected in both ecoregion A and ecoregion B. The cost of protecting overlapping species represented by curve AB is determined by the cost of acting in either ecoregion A or B depending on which is selected for investment. For each parcel of land protected in ecoregion A, the number of species protected includes the endemics represented by curve A and also the overlapping species represented by curve AB (Fig. S1).

**References**
