# Supplementary material for: Protecting Biodiversity when Money Matters: Maximizing Return on Investment
Source: PLoS One. 2008 Jan 30;3(1):e1515. doi: 10.1371/journal.pone.0001515 (PMC2212107; doi:10.1371/journal.pone.0001515)
Supplement: Table S1 — Characteristics of the 39 mediterranean ecoregions: total ecoregion area, projected habitat loss (%yr-1), area of natural and semi-natural habitat, area converted and area protected, cost (US$) per km-2, and information on plant species richness (6), vertebrate (‘Vert’) species richness and endemic richness (9). The number of threatened vertebrate species (IUCN Red List categories critical, endangered, and vulnerable) omits the three extinct vertebrate species in the database. Region codes are as follows: Aus = Australia, Ca&Mex = California and Mexico, SA = South Africa, Chl = Chile, and MedB = Mediterranean Basin. (0.15 MB DOC) [file pone.0001515.s002.doc]

| **Region** | **Ecoregion** | **Area (km²)** | **Projected habitat loss (%yr‾1)** | **Area natural & semi-natural habitat (km²)** | **Area converted (km²)** | **Area protected (km²)** | **Cost (US$km‾²)** | **Plant richness** | **Vert richness** | **Total richness** | **Strict endemic vert richness** | **No. of threatened species** |
| --- | --- | --- | --- | --- | --- | --- | --- | --- | --- | --- | --- | --- |
| Aus | Coolgardie woodlands | 137,226 | 0.00008 | 136,204 | 1,022 | 9,166 | 229,431 | 1,800 | 343 | 2,143 | 0 | 10 |
| Aus | Esperance mallee | 115,460 | 0.00109 | 80,209 | 35,251 | 26,503 | 229,431 | 2,900 | 381 | 3,281 | 5 | 13 |
| Aus | Eyre and York mallee | 60,896 | 0.00542 | 21,791 | 39,105 | 5,776 | 229,431 | 2,000 | 383 | 2,383 | 1 | 7 |
| Aus | Jarrah-Karri forest and shrublands | 10,455 | 0.00371 | 10,264 | 191 | 2,898 | 229,431 | 2,200 | 287 | 2,487 | 3 | 11 |
| Aus | Swan Coastal Plain Scrub and Woodlands | 15,210 | 0.00974 | 8,072 | 7,138 | 1,325 | 229,431 | 2,300 | 358 | 2,658 | 5 | 7 |
| Aus | Mount Lofty woodlands | 23,786 | 0.00926 | 7,779 | 16,007 | 1,622 | 229,431 | 2,000 | 393 | 2,393 | 2 | 10 |
| Aus | Murray-Darling woodlands and mallee | 197,895 | 0.00234 | 98,547 | 99,348 | 24,055 | 229,431 | 1,100 | 500 | 1,600 | 2 | 16 |
| Aus | Naracoorte woodlands | 27,531 | 0.00379 | 10,826 | 16,705 | 1,509 | 229,431 | 1,500 | 374 | 1,874 | 0 | 10 |
| Aus | Southwest Australia savanna | 168,713 | 0.00228 | 83,286 | 85,427 | 7,028 | 229,431 | 1,900 | 457 | 2,357 | 7 | 10 |
| Aus | Southwest Australia woodlands | 46,015 | 0.00333 | 31,765 | 14,250 | 3,120 | 229,431 | 2,000 | 338 | 2,338 | 1 | 12 |
| SA | Albany thickets | 17,135 | 0.00185 | 15,970 | 1,165 | 1,403 | 188,310 | 1,200 | 416 | 1,616 | 2 | 9 |
| SA | Lowland fynbos and renosterveld | 32,764 | 0.00000 | 22,233 | 10,531 | 1,183 | 188,310 | 3,000 | 468 | 3,468 | 10 | 20 |
| SA | Montane fynbos and renosterveld | 45,746 | 0.00000 | 45,379 | 367 | 11,027 | 188,310 | 6,300 | 505 | 6,805 | 22 | 23 |
| Ca&Mex | California coastal sage and chaparral | 36,256 | 0.01499 | 28,392 | 7,864 | 679 | 846,883 | 1,650 | 438 | 2,088 | 6 | 11 |
| Ca&Mex | California interior chaparral and woodlands | 64,617 | 0.00728 | 56,347 | 8,270 | 819 | 1,072,889 | 2,105 | 368 | 2,473 | 2 | 10 |
| Ca&Mex | California montane chaparral and woodlands | 20,403 | 0.00495 | 19,823 | 580 | 2,229 | 1,072,889 | 2,075 | 346 | 2,421 | 1 | 5 |
| Chl | Chilean matorral | 148,383 | 0.00810 | 124,266 | 24,117 | 1,332 | 173,572 | 2,600 | 206 | 2,806 | 25 | 8 |
| MedB | Aegean and Western Turkey sclerophyllous and mixed forests | 133,521 | 0.00698 | 85,845 | 47,676 | 1,311 | 817,770 | 2,500 | 430 | 2,930 | 4 | 21 |
| MedB | Anatolian conifer and deciduous mixed forests | 86,382 | 0.00904 | 48,632 | 37,750 | 523 | 357,549 | 2,400 | 347 | 2,747 | 0 | 13 |
|  |  |  |  |  |  |  |  |  |  |  |  |  |
| **Region** | **Ecoregion** | **Area (km²)** | **Projected habitat loss (%yr‾1)** | **Area natural & semi-natural habitat (km²)** | **Area converted (km²)** | **Area protected (km²)** | **Cost (US$km‾²)** | **Plant richness** | **Vert richness** | **Total richness** | **Strict endemic vert richness** | **No. of threatened species** |
| MedB | Canary Islands dry woodlands and forests | 4,968 | 0.00581 | 4,856 | 112 | 222 | 1,374,981 | 1,130 | 107 | 1,237 | 13 | 4 |
| MedB | Corsican montane broadleaf and mixed forests | 3,633 | 0.00606 | 3,606 | 27 | 0 | 2,148,770 | 1,800 | 169 | 1,969 | 2 | 6 |
| MedB | Crete Mediterranean forests | 8,193 | 0.00000 | 6,829 | 1,364 | 61 | 1,096,338 | 1,600 | 154 | 1,754 | 3 | 4 |
| MedB | Cyprus Mediterranean forests | 9,273 | 0.00998 | 6,531 | 2,742 | 766 | 408,615 | 1,500 | 171 | 1,671 | 2 | 2 |
| MedB | Eastern Mediterranean conifer-sclerophyllous-broadleaf forests | 143,882 | 0.02043 | 101,555 | 42,327 | 649 | 476,185 | 3,200 | 523 | 3,723 | 7 | 26 |
| MedB | Iberian conifer forests | 34,444 | 0.00000 | 18,643 | 15,801 | 1,537 | 1,374,981 | 3,200 | 295 | 3,495 | 1 | 13 |
| MedB | Iberian sclerophyllous and semi-deciduous forests | 298,013 | 0.00000 | 100,884 | 197,129 | 3,223 | 1,363,310 | 2,500 | 351 | 2,851 | 0 | 14 |
| MedB | Illyrian deciduous forests | 40,625 | 0.00384 | 16,813 | 23,812 | 987 | 507,558 | 2,800 | 385 | 3,185 | 1 | 14 |
| MedB | Italian sclerophyllous and semi-deciduous forests | 102,238 | 0.00000 | 47,424 | 54,814 | 496 | 2,475,057 | 3,300 | 363 | 3,663 | 0 | 9 |
| MedB | Mediterranean acacia-argania dry woodlands and succulent thickets | 99,985 | 0.00755 | 76,001 | 23,984 | 56 | 181,943 | 1,600 | 343 | 1,943 | 7 | 18 |
| MedB | Mediterranean dry woodlands and steppe | 292,082 | 0.00346 | 286,372 | 5,710 | 2,890 | 93,498 | 1,200 | 364 | 1,564 | 1 | 17 |
| MedB | Mediterranean woodlands and forests | 358,226 | 0.01058 | 263,173 | 95,053 | 1,760 | 124,398 | 1,500 | 425 | 1,925 | 11 | 21 |
| MedB | Northeastern Spain and Southern France Mediterranean forests | 90,829 | 0.00206 | 49,143 | 41,686 | 1,912 | 1,866,258 | 2,800 | 415 | 3,215 | 3 | 16 |
| MedB | Northwest Iberian montane forests | 57,432 | 0.00000 | 38,991 | 18,441 | 3,937 | 1,336,402 | 1,400 | 329 | 1,729 | 0 | 10 |
| MedB | Pindus Mountains mixed forests | 39,590 | 0.00404 | 25,375 | 14,215 | 2,086 | 750,994 | 4,000 | 343 | 4,343 | 0 | 10 |
| MedB | South Appenine mixed montane forests | 13,088 | 0.00000 | 9,255 | 3,833 | 991 | 2,478,809 | 2,100 | 251 | 2,351 | 0 | 8 |
| MedB | Southeastern Iberian shrubs and woodlands | 2,849 | 0.01872 | 1,243 | 1,606 | 67 | 1,374,981 | 400 | 268 | 668 | 0 | 8 |
| **Region** | **Ecoregion** | **Area (km²)** | **Projected habitat loss (%yr‾1)** | **Area natural & semi-natural habitat (km²)** | **Area converted (km²)** | **Area protected (km²)** | **Cost (US$km‾²)** | **Plant richness** | **Vert richness** | **Total richness** | **Strict endemic vert richness** | **No. of threatened species** |
| MedB | Southern Anatolian montane conifer and deciduous forests | 76,449 | 0.01160 | 56,964 | 19,485 | 1,055 | 407,208 | 4,000 | 387 | 4,387 | 6 | 23 |
| MedB | Southwest Iberian Mediterranean sclerophyllous and mixed forests | 71,109 | 0.00000 | 38,947 | 32,162 | 1,079 | 1,272,474 | 2,600 | 344 | 2,944 | 0 | 12 |
| MedB | Tyrrhenian-Adriatic Sclerophyllous and mixed forests | 85,107 | 0.00000 | 46,511 | 38,596 | 1,867 | 2,378,063 | 3,300 | 327 | 3,627 | 8 | 10 |
