# Supplementary material for: Protecting Biodiversity when Money Matters: Maximizing Return on Investment
Source: PLoS One. 2008 Jan 30;3(1):e1515. doi: 10.1371/journal.pone.0001515 (PMC2212107; doi:10.1371/journal.pone.0001515)
Supplement: Figure S1 — A hypothetical example of the species area curves required to account for complementarity for a biogeographic realm that contains two ecoregions (A and B). Curve A and curve B represent the number of endemic species contained in the respective ecoregions A and B and curve AB represents overlapping species in ecoregions A and B. The circles (O) indicate the amount of area currently protected in each ecoregion. (0.44 MB DOC) [file pone.0001515.s003.doc]

**AB**

**B**

**A**
